# An explainable Artificial Intelligence software system for predicting diabetes

**DOI:** 10.1016/j.heliyon.2024.e36112

**Published:** 2024-08-10

**Authors:** Parvathaneni Naga Srinivasu, Shakeel Ahmed, Mahmoud Hassaballah, Naif Almusallam

**Affiliations:** a Department of Teleinformatics Engineering, Federal University of Ceará, Fortaleza, 60455-970, Brazil; b Amrita School of Computing, Amrita Vishwa Vidyapeetham, Amaravati, 522503, Andhra Pradesh, India; c Department of Computer Science, College of Computer Sciences and Information Technology, King Faisal University, Al-Ahsa, 31982, Saudi Arabia; d Department of Computer Science, College of Computer Engineering and Sciences, Prince Sattam bin Abdulaziz University, Al-Kharj, 16278, Saudi Arabia; e Department of Computer Science, Faculty of Computers and Information, South Valley University, Qena, Egypt; f Department of Management Information Systems, College of Business Administration, King Faisal University, Al-Ahsa, 31982, Saudi Arabia

**Keywords:** Convolutional neural networks, Bi-LSTM, Blood glucose levels, Spectrogram images, Hyperparameters, ROC curves

## Abstract

Implementing diabetes surveillance systems is paramount to mitigate the risk of incurring substantial medical expenses. Currently, blood glucose is measured by minimally invasive methods, which involve extracting a small blood sample and transmitting it to a blood glucose meter. This method is deemed discomforting for individuals who are undergoing it. The present study introduces an Explainable Artificial Intelligence (XAI) system, which aims to create an intelligible machine capable of explaining expected outcomes and decision models. To this end, we analyze abnormal glucose levels by utilizing Bi-directional Long Short-Term Memory (Bi-LSTM) and Convolutional Neural Network (CNN). In this regard, the glucose levels are acquired through the glucose oxidase (GOD) strips placed over the human body. Later, the signal data is converted to the spectrogram images, classified as low glucose, average glucose, and abnormal glucose levels. The labeled spectrogram images are then used to train the individualized monitoring model. The proposed XAI model to track real-time glucose levels uses the XAI-driven architecture in its feature processing. The model's effectiveness is evaluated by analyzing the performance of the proposed model and several evolutionary metrics used in the confusion matrix. The data revealed in the study demonstrate that the proposed model effectively identifies individuals with elevated glucose levels.

## Highlights

• Acquisition and processing of the spectrogram images plotted from the blood glucose levels
• To mechanize the model that can remember the outcomes of the previous state using Bi-LSTM for better precision.
• The machine interpretability in the current study's feature engineering process would assist in determining the feature significance in the decision process.
• Analyzing the model's performance with other contemporary techniques concerning various evaluation metrics.

## 1 Introduction

Diabetes is a chronic condition that has been known for decades. Several instances, however, are detected in their late stages. Diabetes affects one out of every eleven adults worldwide. The World Health Organization (WHO) predicts that by 2040, the number of individuals with diabetes will rise to 642 million, or one in every 10 people [1]. In addition to the four main categories, there are other sub-categories. A person develops type 1 diabetes, also called insulin-dependent Type-2 diabetes, when the insulin production in the pancreas stops working. Insufficient insulin production by the body characterizes type-2 diabetes. People over 40 are more likely to have this problem. Gestational diabetes (GDM) occurs most often during pregnancy. prediabetes which develops whenever blood sugar levels are elevated but not severe enough to qualify as type-2 diabetes, is the fourth main category [2]. As a result, early diagnosis and appropriate treatment of diabetes may help prevent impediments and reduce the risk of serious health consequences. Several bioinformatics researchers have taken on this problem by building methods and tools for diabetes prediction. Several body composition changes may aid the early identification of Type 2 diabetes. Maintaining a good dietary cycle is critical in minimizing the risk of developing diabetes and its complications. Our presentation emphasizes earlier research investigations on the diagnostics of Type 2 diabetes and early identification of diabetes based on real-time glucose data [3].

Wearable, non-invasive, and intelligent patient monitoring technologies are in high demand. As a result of previous research, wearable glucose monitoring devices based on various technologies have been developed. It was shown that glucose concentrations could be measured using plasmonic, carbon nanotube, and fluorescence sensors [4,5]. Lengthy calibration procedures, large size, and autofluorescence sensitivity were some of these approaches’ drawbacks [6,7]. There is a necessity for a model that could precisely diagnose the abnormal glucose level in the body and timely notify the primary healthcare centers to provide the appropriate medication. Due to life obligations, financial restraints, and an insufficient number of medical practitioners, particularly in rural and developing regions, communities may not seek regular health exams, resulting in late diagnosis of illnesses that can cause serious sickness if neglected or treated late [8].

The main purpose of our XAI modeling is trustworthiness since we intend to maximize a Convolution layer more reliably so that possible mishaps may be avoided. As a result, our XAI model increases the trustworthiness of a Classification algorithm by employing feasibility analysis, with some sample images and visible explanations about the decision mechanism [9]. It is a technique for estimating the impact of every input parameter. First, we assign a particular value to each input variable and feed the changed input vector into the model. Resultantly, one can calculate how much the output differs from the model's output vector whenever the input is the original input data. So, we can observe which pieces of the input greatly affect the output vector, and hence which portion of the input causes the model to determine correctly or incorrectly [10]. Some of these things may result in a major mishap if the decisions being made by these systems are incorrect. At the same time, the evaluator can approximate the total number of neurons used in deep learning decision-making models but is unsure of the exact number for each neuron in these models.

The current study primarily focuses on real-time monitoring of diabetic patients to track abnormal glucose studies. The existing studies acquire the blood glucose levels as a numerical entity and alarm the caretakers when they experience an abnormal glucose value, rather in the current study, the signal data is converted to the spectrogram images, and those spectrogram images are analyzed to determine the glucose levels as the signal to numerical conversion is prone to errors. Moreover, determining the abnormal glucose level from signal data is less effective [11]. Analyzing the spectrogram images to determine the glucose levels using a deep learning model is the novelty aspect of the current study [12,13]. Analyzing the spectrogram images for glucose tracking through the deep learning model is a first of its kind. Bi-LSTM [14] would assist in precisely identifying the abnormal glucose levels. The XAI technology assists in fostering trust and enhancing transparency inside Artificial Intelligence (AI) systems, particularly in high-stakes sectors such as healthcare. This can help individuals make more informed decisions, comprehend the rationale behind suggestions or actions, and subsequently take suitable measures based on such insights.

Due to the rising percentage of geriatric people in the population, there is an expanding demand for assisted-living options that allow senior citizens to maintain their independence in their own homes for an extended period of time, thus decreasing their reliance on caretakers. The proposed methodology is largely motivated by assisted technology, which continuously monitors individuals for abnormal glucose levels. The primary objective of real-time monitoring is to make immediate adjustments to treatment protocols in order to optimize outcomes. Continuous monitoring promotes patient engagement in condition management through the provision of individualized, actionable information regarding their health in real-time [15]. The utilization of wearable technology permits healthcare professionals to monitor patients’ health status remotely, eliminating the necessity for frequent in-person consultations, and thereby reducing the cost of healthcare services. The proposed methodology would allow the monitoring of multiple patients simultaneously.

**Fig. 1:**
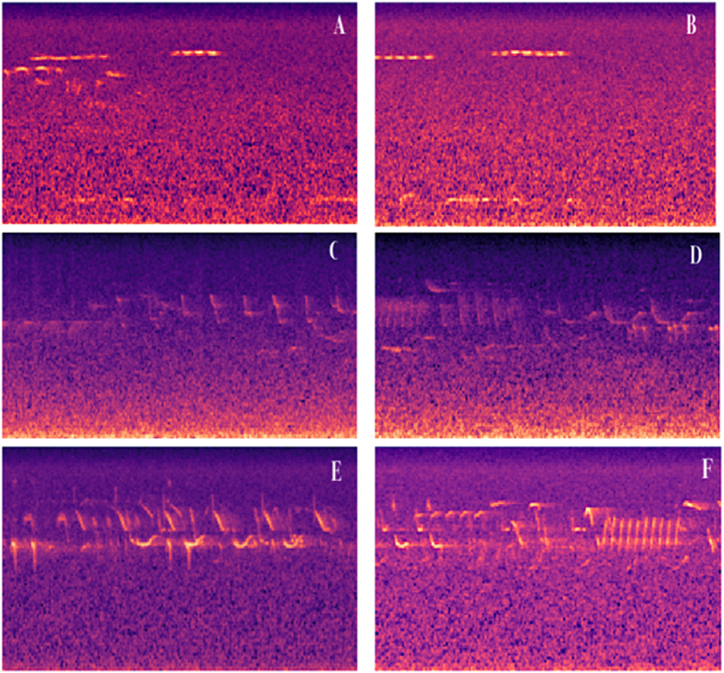
Spectrogram images represent the blood glucose levels.

The blood glucose levels are acquired through the GOD strips placed over the human body. Later, the signal data is converted to the spectrogram images, which are classified as low glucose, average glucose, and abnormal glucose levels. The labeled spectrogram images are then used to train the model for individualized monitoring. Multi-resolution wavelet analysis decomposes the mixed signal into sub-signals of diverse frequency bands. Uses wavelet transform's breakdown and rebuilding operations to split signals into discrete frequencies and eliminate high-frequency band noise from the output signal. A signal is reconstructed using low-frequency components. The short-time Fourier transform (STFT) converts the signal to the spectrogram images. Fig. 1 presents the spectrogram images used in the model's training and validation process. Where sub-figure (A) and (B) present the spectrogram images of a patient with lower glucose levels. The sub-figures (C) and (D) denote the spectrogram images of normal glucose levels. The sub-figures (E) and (F) denote the spectrogram images of abnormal glucose levels. The spectrogram images are classified based on the features mapped with the glucose level. In the current study, the spectrogram images with normal glucose levels are considered as one class, and the spectrogram images with abnormal glucose levels are considered as another class.

This research compared several machine learning-based approaches for predicting diabetes and reported our findings in a comparative analysis. The following is a rundown of the many contributions that this research has made.

• To discuss and outline briefly various cutting-edge technologies in diabetes prediction.
• Acquisition and processing of the spectrogram images plotted from the blood glucose levels.
• To mechanize the model that can remember the outcomes of the previous state using Bi-LSTM for better precision.
• The machine interpretability in the current study's feature engineering process would assist in determining the feature significance in the decision process.
• Analyzing the model's performance with other contemporary techniques concerning various evaluation metrics.

The remaining sections of the paper were arranged as follows, Section 2 Section 3 discusses the process of spectrogram image generation and pre-processing, which is necessitated for precise classification of the spectrogram images. Section 4 discusses the proposed XAI framework for feature engineering. Section 5 discusses the CNN with Bi-LSTM for classifying the abnormal glucose levels from the spectrogram images. Section 6 provides the results obtained from experimentation and their statistical analysis, and Section 7 gives the conclusion and the future scope.

## 2 Literature review

Automated data-driven methods do not need an understanding of the structural and physiological processes since they utilize the individual's collected data. An individual's degree and duration of physical activity, as well as their experience with hypoglycemia and the function of their pancreas, influence blood glucose dynamics in people with diabetes. Previously, models with input parameter combinations accounted for these aspects were examined. In recent years, several ML and DL-based models for diabetes prediction have been designed, including Logistic Regression (LR), XGBoost (XGB), Multilayer Perceptron (MLP), Support Vector Machine (SVM), Artificial Neural Network (ANN), Linear Discriminant Analysis (LDA), Quadratic Discriminant Analysis (QDA), Decision Tree (DT), Random Forest (RF), AdaBoost (AB), J48, k-Nearest Neighbours (k-NN), random forest (RF), and Light Gradient Boosting Machine (LGBM) [16,17]. Various feature reduction and cross-validation strategies and the process for handling the missing data and discarding outliers have been used to boost the efficiency of ML and DL models [18,19].

Lin et al. [20] evaluated Naive Bayes, SVM, and ANN classifiers in their study utilizing a diabetic dataset. They conducted a weighted investigation in which most votes determined the likelihood of diabetes prognosis. Finally, they determined that combining models improves classification accuracy compared to single algorithm-driven models. On the diabetes dataset, Kandhasamy et al. [21] suggested a predictive analytic model employing J48 (C4.5), k-NN classifier, RF classifier, and SVM. They planned that the J48 method would perform better than others before pre-processing data, with 73.82 % accuracy. Still, KNN and Random Forest would perform a much better way of performing the pre-processing. Dey et al. [22] employed ensemble classification mechanisms like SVM, KNN, Naive Bayes, and ANN with Min-Max scaling (MMS) over a structured dataset like Pima. The ANN models with MMS have a greater accuracy of 82.35 percent than the other four methods. Sonar and JayaMalini [23] built a model to effectively identify diabetes patients through machine learning methods, including DT, ANN, NB, and SVM classification algorithms, where the DT has obtained an accuracy rate of 85 %. Beloufa and Chikh [24], report an 86.13 % accuracy associated with Support Vector Regression (SVR) and an ANN-based algorithm for diabetes identification.

A study on the Gaussian Hidden Markov Model (GHMM) method has attained 85.69 % accuracy [25], and a Deep Extreme Learning Machine (DELM) oriented prediction technique has achieved 92.8 % accuracy [26]. Sanakal and Jayakumari [27] utilized FCM clustering and SVM for the precise classification of diabetic records over the PIMA dataset records and achieved 94.3 % accuracy. Compared to earlier efforts, Hasan Temurtas et al. [28] utilized the Levenberg–Marquardt (LM) technique for the diabetic diagnostic model. It has obtained an accuracy of 82.37 %. Another study based on CNN-LSTM for diabetes classification has achieved 90.9 % test accuracy. CNN model over the 5-fold cross-validation accuracy was 93.6 %, whereas CNN with LSTM's 95.1 %. In the study on diabetes prediction [29], the Authors gathered 289 cases and 13 characteristics from the Khulna Diabetes Center. In our analysis, we apply Logistic Regression (88 %), XGB (86.36 %), and Random Forest (86.36 %). Saha et al. [30] employed ML methods like neural networks (NN), SVM, and Random Forest to predict diabetes. Preprocessing techniques like imputation, data normalization, and feature selection techniques like principal component analysis were employed. The Neural Network was the best and most consistent model with setup settings for 100 epochs with a batch size of 10. It obtained an accuracy of 80.4 %. Wei et al. [31] deployed a DNN model for preventing type 2 diabetes and observed an accuracy of around 77.86 % percent. After performing the scaling, the ReLu activation over the Nadam optimizer was over 100 epochs with a batch size of 10. Smartphones and wearable sensor data were utilized to construct a disease pre-warning system [32].

This system classifies health issues using data analysis, ontologies, and deep learning. However, all the models mentioned above rely on pre-existing data to predict diabetes in patients. The models need to be robust to handle the real-time data, and they need a tremendous volume of data to train them to attain considerable accuracy. The proposed model is designed to monitor the real-time blood glucose level and alarm the individuals for timely medication or appropriately notify the caretakers. There is another concern related to data imbalance in diabetes prediction. Particularly big is the data of blood glucose measurements taken at normal levels which is considered as the majority class, e.g., hypoglycemia, and then the dataset of blood glucose measurements taken over the specific symptoms, which is considered as the minority class. An unbalanced dataset leads to a model with a biased outcome, which means that the precision of the minority class is substantially worse than that of the class label. This is another pivotal reason for migrating toward real-time blood glucose analysis, which is the present research problem. Most often, the RF and SVM models were used, with the DT model being utilized in conjunction with the RF model in certain circumstances. However, performance metrics revealed that the DT model frequently scored worse than the RF model [33]. The SVM model employed a variety of kernel functions because of the variety of models that were utilized. Regression models such as SVR and linear regression are often used. The LR model was used rather than the linear regression model because of the categorical nature of the dataset. The multinomial LR model was utilized since the intended feature classification output had not been binaries but three-class (low, normal, and high blood glucose level) problems to be evaluated [34].XAI technology is used in various healthcare domains to make the decision process evident in various healthcare applications [35,36]. The existing studies on XAI for healthcare have proven to outperform in efficiency and the ability to explain the decision model. A study on XAI for image analysis through a deep learning model [37]. The performances of various state-of-the-art models used in assessing Type-2 diabetes using ML and clinical approaches (CA) are presented in Table 1.

**Table 1:** State-of-art techniques used in Type-2 diabetes Prediction.

Technique	Algorithm	Data Type	Overall Accuracy	Remark
ML Technique Ahamed et al. [2]	LR	Tabular Data	75.2 %	Used in assessing diabetes from pre-existing data using feature selection.
	XGB		83.3 %	
	GBC		94.1 %	
	DT		94.4 %	
	RF		94.8 %	
	LGBM		95.2 %	
ML Technique Kulkarni et al. [38]	XGB	ECG data	96.8 %	The study is limited to the early detection of diabetes.
ML Technique Ahmed et al. [39]	NB	Tabular Data	86.1 %	Prediction of diabetes from the pre-existing tabular data by feature selection.
	DT		96.8 %	
	GBC		91.0 %	
CA Approach Saha and Saha [40]	RCT	Real-time blood sample data	95.0 %	The approach is invasive and needs frequent finger pricking.
ML Technique Shen et al. [41]	NB	IoT and Embedded systems for real-time data	84.1 %	The approach is invasive and needs finger pricking.
	J48		99.7 %	
	LR		86.0 %	
	RF		99.6 %	

## 3 Spectrogram image generation and pre-processing

The spectrogram images are generated from the real-time blood glucose levels observed from the sensors connected to the human body. Blood glucose levels may be seen as a time series signal, with time over the x-axis and blood glucose concentration over the y-axis. Short-time Fourier transforms a series of Fourier transformations performed on a windowed input signal. When a signal's frequency components vary over time, the STFT offers time-localized frequency information, whereas the conventional Fourier transform delivers frequency components averaged across the whole signal period. Using multi-resolution wavelet analysis, the chaotic signal is broken down into component signals in their unique frequency ranges. The wavelet transform's decomposition and reconstruction techniques are used in signal denoising to separate the noisy signal into its component bandwidths and eliminate the noisy high-frequency band. The low-frequency components are used to reconstruct a signal. Short Fourier transformations can be used to modify this signal to provide a considerably improved frequency distribution throughout time. Equation (1) demonstrates the converted signal identified by the variable $O_{s}\left(\kappa \right)$.

(1) $$O_{s}\left(\kappa \right)=\frac{1}{2\pi }\int_{-\infty }^{\infty }e^{-i\kappa t}I_{S}\left(\lambda \right)\omega_{s}\left(\lambda -t\right)d\lambda$$

From the above Equation, the variable $I_{S}\left(\lambda \right)$ denotes the original input signal to the STFT function, operated through the window $\omega_{s}\left(\lambda \right)$, where the length of the window impacts total resolving power concerning time and frequency. The STFT may be considerably enhanced by adding a window to a non-stationarity input. With this window, the frequency obtains a new temporal dimension. The intensity magnitude is determined by Equations (2), (3), which assumes a homogenous signal filter band.

(2) $$\Theta \left(s\right)=\omega \times \sum_{p=-\infty }^{\infty }m\left(p\right)\times \left(\alpha -p\right)$$

(3) $$f\left(\alpha ,\omega \right)=\Theta \left(s\right)\times \sum_{p=-\infty }^{\infty }e^{-c\omega p}$$

From the above Equation, the variables α and ω designate the time and frequency components in the signal processing. The variable m represents the related window function with a zero-centered interval p.

The change in glucose levels may manifest as changes in the frequency content of the signal, which would be visible as variations in the spectrogram. For example, higher glucose levels might correspond to increased activity in certain frequency bands, leading to more intense areas in the spectrogram. Windowing functions may emphasize certain frequency elements in the spectrogram. To emphasize low-frequency components, the window functions with a peak in the frequency domain. One such example is the Gaussian window. The mathematical definition of the Gaussian window $\omega_{g}\left(x\right)$ is shown in Equation (4).

(4) $$\omega_{g}\left(x\right)=e^{-\frac{x^{2}}{2\sigma^{2}}}$$

σ is a parameter that determines the width of the main lobe. A bigger σ number creates a broader main lobe, emphasizing lower frequencies. The filtering method is employed to extract low-frequency components from spectrogram images. The mathematical expression for the output Y(f) of a low-pass filter applied to the spectrogram image X(f) is as shown in Equation (5).

(5) Y(f)=X(f)×H(f)

From the above equation, H(f) represents the low-pass filter's frequency response. This filter selectively passes sounds below a predetermined cutoff frequency, highlighting low-frequency elements in the spectrogram.

The dataset employed in this study is processed for the class imbalance issue, where numerous spectrogram images of abnormal glucose instances are present, which slows down the model training process and impacts the model's accuracy. The modified entropy loss function is used in the current study to address this issue. By lowering the weight of easily classifiable samples, this function may improve the model's focus on the difficult-to-classify samples in the training phase. The modified cross-entropy loss function formula is shown in Equation (6).

(6) $$L_{f}=‐\text{tx}{\left(1‐x^{\prime }\right)}^{\eta }\;\log\;x^{\prime }‐\left(1‐x\right)\log\;\left(1‐x^{\prime }\right)$$

From the above equation, the variables t target balancing the positive and negative data samples concerning loss value. The variable η parameter directs the model's attention to indistinguishable data samples, improving the model's overall classification accuracy [9]. Furthermore, the quality of the spectrogram images is being enhanced for better processing and recognition of the image features. The spectrogram images are processed using the Otsu-based Adaptive Weighted Bilateral Kernel (AWBK) for enhanced contrast. AWBK approach uses the Otsu thresholding approach. The non-linear kernels maintaining the target's boundary information (edge) may reduce noise by noisy aggregating sites in the picture concerning pixels directly around it. Assuming the deformed pixel is reconstructed using the contra-harmonic average of pixel intensities in the spectrogram image's noisy region. It's generally assumed that pixels that are close together will have high pixel intensities and that the weight will be significant. The weight's significance decreases as we step away from the pixel's centroid and becomes negligible far away [36]. In which the average pixel intensity of the surrounding pixels has been maintained, and the noise has been disregarded, the noise would be weakly correlated. When a low pass kernel is used to compute the average of the pixel intensities during the image enhancement stage, it will smoothen the picture, i.e., blur the area of the image, but the bilateral edge kernel. The texture information is kept just for processing convenience. To utilize the bilateral kernel, the pixel's degree of belongingness $D_{x}$ and maximum likelihood $m_{s}\text{,}$ A Gaussian distribution function is used over the centroid. The weighted sum of image pixels p of kernel size k is determined using Equation (7).

(7) $$B_{f}=\frac{1}{m_{s}}\sum_{K}D_{x}\left(\left\|a-b\right\|\right)m_{s}\left(\left\|i_{a}-i_{b}\right\|\right)$$

From the above equation, the variables a and b denote the corresponding neighboring pixel, and similarly, the variables $i_{a}$ and $i_{b}$ denotes the intensities of both the pixels a and b, respectively. The variable $D_{x}$ is used to assess the corresponding pixel's belongingness. The variable $m_{s}$ denotes the contra-harmonic mean calculated as shown in Equation (8).

(8) $$m_{s}=\frac{\alpha_{1}^{2}+\alpha_{2}^{2}+\alpha_{3}^{2}+\ldots +\alpha_{n}^{2}}{\alpha_{1}+\alpha_{2}+\alpha_{3}+\ldots +\alpha_{n}}$$

The thresholding strategy, such as Otsu, reduces the interclass correlation variance by evaluating the weighted probabilistic average across several classes that may be assessed using the following process for a spectrogram image. $\omega_{p}$ and $\omega_{q}$ the probability weights of categories m and n are indeed categorized using an approximation of the threshold t as shown in Equation (9).

(9) $$V\left(t\right)=\omega_{p}v_{p}\left(t\right)+\omega_{q}v_{q}\left(t\right)$$

**Fig. 2:**
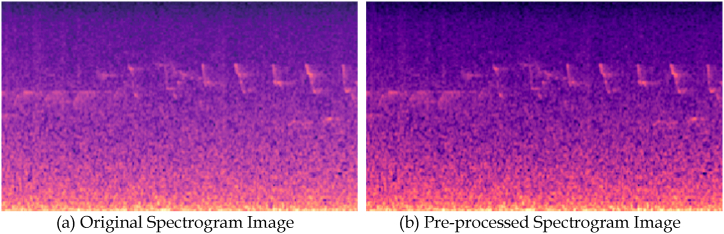
Image representing the original and pre-processed spectrogram image.

**Fig. 3:**
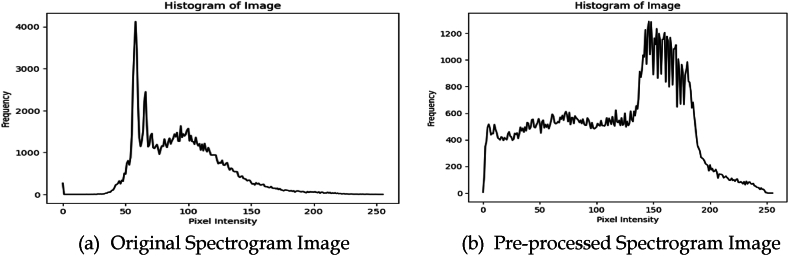
Histograms of original and pre-processed images.

On pre-processing the spectrogram images, the quality of images has tremendously improved, and they would assist in better classifying the spectrograms by abnormal glucose levels. The spectrogram images after the pre-processing are shown in Fig. 2, where the sub-figure (a) represents the original spectrogram image and (b) represents the pre-processed image. The histogram of the original image and the pre-processed image is shown in Fig. 3 for understanding the impact of the pre-processing technique, where the sub-figure (a) is corresponding to the original spectrogram image and (b) is corresponding to the pre-processed spectrogram image. Where the sequences are distributed across the image rather than accumulating towards a confined range of frequencies as in the original spectrogram image.

The spectrogram images are generated from the glucose levels at the instance of time, but the long-term dependencies are not considered. Long-term dependencies are desired for better precision of the classification outcome. There is a possibility of Temporal Resolution Loss (TRL) [42] and Dynamic Range Compression (DRC) [43], which leads to a considerable loss in the spectrogram image generation process. The current study was limited to addressing both the challenges that are discussed above.

## 4 XAI-driven feature engineering

**Fig. 4:**
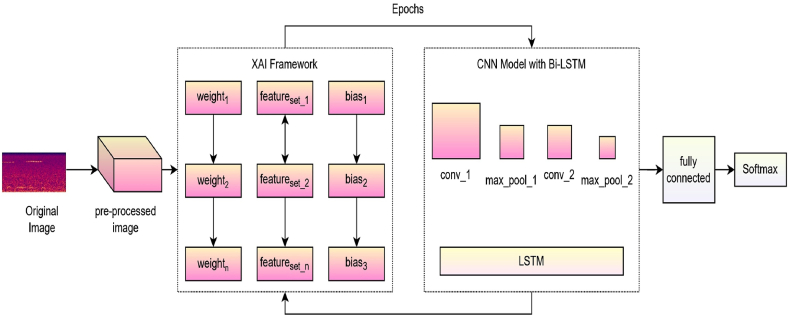
Block diagram of the proposed XAI-driven CNN with Bi-LSTM model.(11)$L_{train}=\frac{1}{f_{t}}\sum_{\nu =1}^{f_{t}}f_{x}\left(\theta \right)\times \omega_{y}$

The weights of neurons cannot be immediately interpreted as information, and the size and sensitivity of activations are not suitable indicators of a neuron importance for a specific task. As we know, the deep learning models are still not fully understood. A full examination of the underlying architecture, procedures, and predictable statics that machine-interpretable and explainable models can obtain. Diagnostic data is analyzed using XAI techniques to better display the outcomes of a machine learning system [44]. Tasks like feature extraction and feature extraction with feature weights fall within the purview of feature engineering. The XAI model is then used to optimize the loss function and initialize the feature weights. The elements considered more important in the assessment phase are given greater weightage than the rest. Transparency is anticipated to ensure that the model is visible. The corresponding figure for XAI-driven feature engineering is shown in Fig. 4.

Each iteration updates the weights in each neural network layer. The performance of the classification algorithm depends on initial weights, biases, and activation functions. Transparency of weight approximation functions helps reveal the Operational Procedure Model (OPM). In weight initialization, more important traits are given higher weights and are later optimized [37]. Equation (10) shows the weight initialization process for better model transparency.

(10) $$\omega =\sum_{x=1}^{p}\sum_{y=1}^{q}\left|\omega_{xy}\times \omega_{yo}\right|$$

From Equation (7), the variable $\omega_{x,y}$ designates the association among the weights of the input node x and the corresponding hidden node y. Similarly, the weight $\omega_{yo}$ represents the associated weights among the hidden node y and the corresponding output node represented by o. Over iterations, weights are tweaked for greater performance. Considering model-specific characteristics and loss functions optimizes weight. The loss caused over the training phase is denoted by the variable provided in Equation (11) using the variable $L_{train}$.

The variable $\omega_{y}\text{,}$ represents the feature's weight, which is being updated to use the validation and training component ${\left\{\omega_{y}\right\}}_{y=1}^{f_{t}}$ .Equation (12) determines the loss function linked with the feature weights from data used for training, which should be the smallest.

(12) $$\omega^{\prime }=\frac{1}{f_{t}}\sum_{y=1}^{f_{t}}f_{y}^{\prime }\left(\theta \times \omega \right)$$

Throughout the training phase, the weight is optimized, the number is finetuned, and the values are advised to be higher than zero for an effective diagnostic model [2]. The accurate assessment depends largely on feature selection, and the feature selection method is desired to be transparent for any trustworthy system. The feature set that is thought useless is deleted from deliberation in the assessment process. In contrast, the feature set presumed to be significant is made more obvious [45]. Equations (13), (14) are then used to update the fitness for the most important feature.

(13) $$f^{\prime }=f_{x}+\eta \left(\left(t_{u}-t_{l}\right)\vartheta +t_{l}\right)\gamma \geq 0$$

(14) $$f^{\prime }=f_{x}-\eta \left(\left(t_{u}-t_{l}\right)\vartheta +t_{l}\right)\gamma <0$$

From the above Equations, the variables $t_{u}$ and $t_{l}$ denotes the upper and the lower threshold values respectively. The other coefficients, like η,ϑ,γ, are the balancing factors, whose values are calculated using Equations (15), (16).

(15) $$\eta =2e^{-{\left(\frac{4m}{m_{t}}\right)}^{2}}$$

(16) $$\vartheta =\delta_{x}$$

Where in the above equations, the variable m denotes the current iteration and the $m_{t}$ denotes the sum of all epochs. The variable $\delta_{x}$ denotes the chaotic gradient of the current epoch. The value of g is likely determined during the training or optimization phase of the model. It may be adjusted based on empirical results, experimentation, or domain knowledge. Suppose we have a loss function L(γ) that we want to minimize with respect to the variable g. This loss function could represent the error of our model on a training dataset or some other measure of performance. Set $g=g_{0}$ with a learning rate α that controls the step size in each iteration. Determine the gradient of the loss function relative to g is designated by $\frac{\partial L}{\partial g}$. The g values are updated as shown in Equation (17).

(17) $$g^{\prime }=g-\alpha \frac{\partial L}{\partial g}$$

In the above equation, the $g^{\prime }$ is the new updated value of g that is determined based on the old value. Equation (18) determines the fitness value for assessing the feature set.

(18) $$f\left(x\right)=\omega \times acc+\left(1-\omega \right)\times \left(1-\frac{I_{gain}}{{tot}_{f}}\right)$$

The acc variable denotes the accuracy associated with the current epoch, $I_{gain}$ is the associated information gain, and the variable ${tot}_{f}$ denotes the total number of associated features.

## 5 CNN with Bi-LSTM for spectrogram classification

The integration of CNN and Bi-LSTM models in this approach effectively exploits the respective advantages of each architecture, enabling the simultaneous extraction of spatial characteristics and temporal dependencies within the dataset. CNN has shown its efficacy in capturing spatial characteristics present in images, including but not limited to edges, textures, and forms. On the other hand, Bi-LSTM networks excel at capturing temporal patterns and dependencies that exist within sequences. Also, the Bi-LSTM models avoid the overfitting issue.

**Fig. 5:**
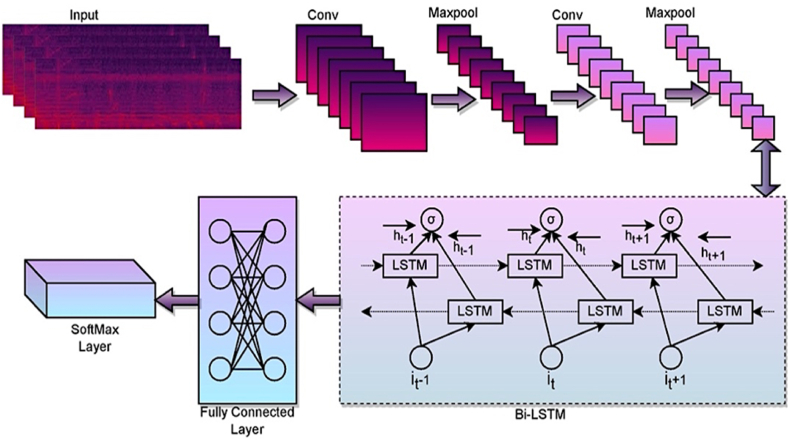
The layered network diagram of the CNN with the Bi-LSTM model**.**

Convolutional neural network is a type of Multilayer perceptron (MLP). The following are some of how they are analogous to neural networks: The neurons are updated with the weights and biases that need to be acquired through training. There is some input reaching each neuron. After that, a dot product implementation is carried out, which may or may not be followed by a non-linear function. Initial applications of CNNs were found in image processing, where the network would take in unprocessed image pixels on one end, convert them via a series of hidden layers, and then produce class scores on the other. The major layers of CNN may be broken down into three categories. The convolutional, pooling, and fully connected layers long with rectified linear activation (ReLU) functions are the layers included in the network [38]. Finally, the softmax layer is used in probabilistic measures to determine the belongingness of the class. In this case, CNN uses one-dimensional time-series data organized in order of successive time instants. A CNN's primary notion is to extract local characteristics using upper-layer data and transmit it to subsequent layers for more significant features. The layered network architecture of the CNN with the Bi-LSTM model is illustrated in Fig. 5. The size of the input spectrogram image in the current study is 224×224. The original spectrogram images are resized before they are fed as input to the CNN model.

The convolutional layer has kernels for determining associated feature maps. Those kernels roll out over the input using stride to create integer output volume dimensions. The input volume shrinks when the convolution is utilized to stride [39]. Padding with zeros is needed to pad the input vector with zeros and keep its low-level dimensions. The equation for the convolutional layer over the input vector v over the 2D kernel k of size m×n is shown in Equation (19).

(19) f(p,q)=∑∑s(p+m,q+n)×k(m,n)

ReLU layers boost non-linearity in the feature maps [41]. ReLU calculates activation with zero thresholds, as shown in Equation (20).

(20) f(r)=max(0,l)

The pooling layer down samples a given input dimension to limit the number of parameters. The most frequent approach is max pooling, which creates the highest values over the input region. The FC layer serves as a classifier, making decisions based on the characteristics gathered from convolution and pooling. To find the probability distribution over labels, use the softmax function. The likelihood of classifying that use the softmax function. The equation for softmax is given in Equation (21).

(21) $$p\left(m^{\left(x\right)}=y|n^{\left(x\right)};\theta \right)=\frac{e^{\left(\theta_{y}^{T}\right)n^{\left(x\right)}}}{\sum_{k=1}^{z}e^{\left(\theta_{y}^{T}\right)n^{\left(x\right)}}}$$

For example, when a convolution accompanies a normalizing procedure, the batch normalization settings were examined to speed up training. All of the batch normalization settings are constant. The convolution process may be used to include these constant parameters. In the training process, the batch normalization $b_{n}$ is assessed through the standard deviation σ and mean m over the input component i, with an assumption that for each mini-batch $m_{b}$, the variance should be 1 or the average should be 0.

(22) $$b_{n}=\sigma \times \frac{i-\Upsilon }{\sqrt{{\left(m_{b}\right)}^{2}+\kappa }}+m$$

(23) $${m_{b}}^{2}=\frac{1}{p}\;\sum_{x=1}^{p}{\left(i-\Upsilon \right)}^{2}$$

(24) $$\Upsilon =\frac{1}{p}\sum_{x=1}^{p}i$$

From Equations (22), (23), (24), the variable κ denotes the hyperparameters used in finetuning the training time of the model. Resultantly, the learning phase is stabilized, and the sum of epochs in the training phase required to build neural networks is significantly reduced.

### 5.1 Integration on Bidirectional-LSTM

The Bi-LSTM component consists of an LSTM component that could function in either of the directions, allowing it to include previous and prospective context summaries. Bi-LSTM would learn long-term dependencies by normalizing the redundant background information [46]. The findings have demonstrated outstanding efficiency in sequential modeling issues and are widely utilized in text categorization. The Bi-LSTM framework, when compared to LSTM architecture, includes dual concurrent layers that move in different directions by forwarding and backward passes to capture interdependence in other contexts. It takes the features created by the CNN process and identifies them from the final hidden layer. Bi-LSTM provides feasibility in accessing previous and following context data, and the information produced by Bi-LSTM may be thought of as two distinct futuristic representations. The CNN features are supplied into a Bi-LSTM model, which generates a sequence characterization. This final feature map is put into an attention layer, selecting the characteristics strongly associated with the final classification. The forward LSTM network learns from prior data in the forward move, whereas the backward LSTM learns from forthcoming values in the opposite direction. In the final layer, the learned information from the previous concealed states is merged [46]. Equations (20), (21) describe the processes carried out in the Bi-LSTM unit identified by BiLSTM over the forward LSTM component $\vec{f\left(x_{t}\right)}$ and the backward component $\overset{⃖}{f\left(x_{t}\right)}$.

(25) $$\vec{f\left(x_{t}\right)}=\sigma \left(\omega_{1}p_{t}+\omega_{2}p_{t‐1}\right)\times \tanh\;\left(k_{t}\right)$$

(26) $$\overset{⃖}{f\left(x_{t}\right)}=\sigma \left(\omega_{3}p_{t}+\omega_{4}p_{t‐1}^{\prime }\right)\times \tanh\;\left(k_{t}^{\prime }\right)$$

(27) $$BiLSTM=\omega_{5}\vec{f\left(x_{t}\right)}+\omega_{6}\overset{⃖}{f\left(x_{t}\right)}$$

From Equations (25), (26), (27), the variable $p_{t}$ is the input at the time stamp t, over the weights $\omega =\left\{\omega_{1},\omega_{2},\omega_{3},\omega_{4},\omega_{5},\omega_{6}\right\}$ associated with the gates. An L2 regularize, and an activation function tanh are used to complete normalization, which may assist in preventing over-fitting. When the network is run, the following layer combines vectors of size α×β, where the variable β denotes the neurons in each LSTM unit.

### 5.2 Batch normalization

Batch Normalization (BN) is often used to enhance the efficiency of the training process, which influences the accuracy of the prediction. When tuning the learning rate to be high, most neural networks may explode or vanish. In such a context, batch normalization is to solve these issues. Normalizing activation throughout the network helps to prevent modest changes to parameters that might result in these parameters amplifying or disappearing so abruptly. Since implementing batch normalization and ReLU, classification accuracy has dramatically increased. Batch normalization led to faster training, shorter training and testing times, and decreased sensitivity to initialization in our trials. Before the activation function, batch normalization is implemented, which is represented as shown in Equation (28).

(28) n=ωp×β

The weight is denoted by the variable ω, and the variable β denotes the associated bias. The normalization is performed over the input component p, where Normalization satisfies the convolution property, in which various parts of the same feature map are normalized in the same manner at different instances.

### 5.3 Dataset description

**Fig. 6:**
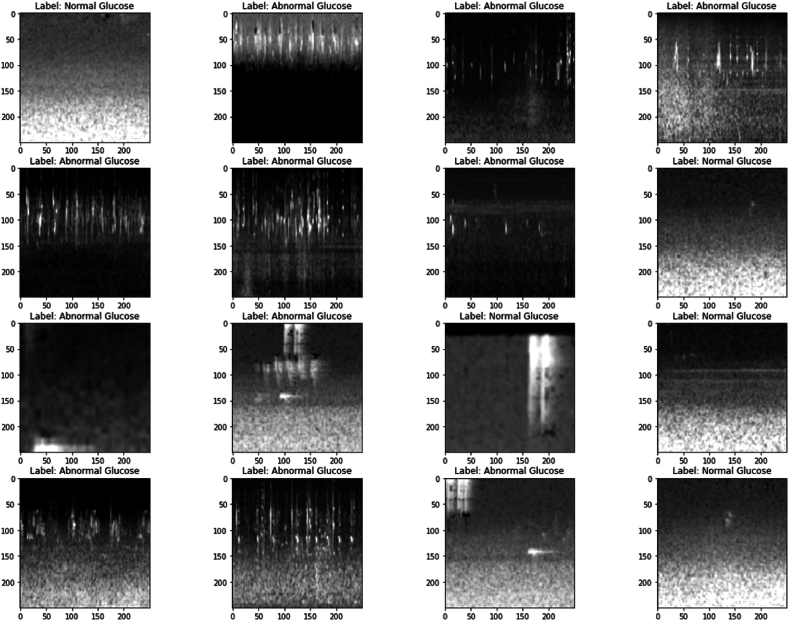
Images with class labels used in model building.

This information comes from the HR waves created by the GOD sensors connected to the human body that monitor glucose levels. The spectrogram images are generated using a short-time Fourier transform. The glucose levels are manually tagged as −1, 0, and 1 to properly identify the spectrogram images. After the images have been tagged, the CNN with the Bi-LSTM model is trained on the data. To make the model simple to implement and evaluate, the spectrogram images are considered as two classes, i.e., the images with normal and abnormal glucose levels. The total dataset consists of 6735 spectrogram images, among which 1347 images are used to identify people with aberrant glucose levels, of which 2806 are related to normal glucose levels (GL normal), 2658 samples are high glucose levels (HG), and 1271 are linked to low glucose levels (GL lows) (abnormal GL). The entire dataset comprises 80-20 training and testing sets, respectively. The same dataset images are shown in Fig. 6. The images are randomly picked from the dataset and shown in a 4×4 grid.

### 5.4 Hyperparameters

The hyperparameter choices for every model used in the tests are described in this section. Determining the best hyper-parameters is difficult and changes depending on the dataset's features [47,48]. For that purpose, we ran multiple experiments to determine which hyper-parameters would provide the highest performance outcomes for the classifiers. Hyperparameters of the CNN and Bi-LSTM layers are presented in Table 2. It is not significant that data points were zero-padded before processing them as input for the deep neural networks, ensuring that all input vectors have the same size. The feature vectors are supplied to a fully-connected layer with 64 nodes in the CNN as a baseline model. A ReLu function and the norm approach train and regularize the dense hidden layers. The associated weights are then driven to the output layer using a sigmoid activation function, which calculates the final classification probabilities. During the training phase of the model, the Adam algorithm is optimized with a predefined initial learning rate of 0.00001, and loss minimization of the cross-entropy mechanism is used [49]. The training and testing instances are obtained from the same image dataset with a proportionate split of the samples. The validation set used to update and fine-tune the model and update the hyperparameters is an integral part of the testing samples.

**Table 2:** Details of the hyperparameters.

Hyperparameter	CNN + Bi-LSTM
Learning Rate	0.00001
Kernel	4
Number of filters	128
Stride	2
Activation Function	ReLu (CNN) and Tanh (Bi-LSTM)
Number of Epochs	30
Dropout Factor	0.3
Batch Size	64
Optimizer	Adam
Max_Pooling	2×2
Nodes in fully connected layer	40
Nodes in the softmax layer	3

**Fig. 7:**
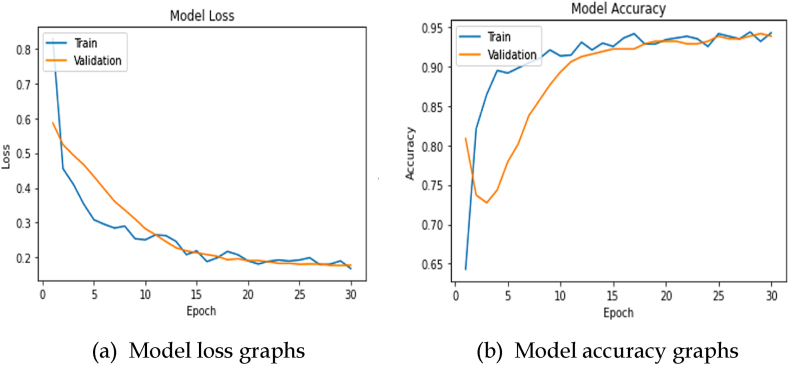
Hyperparameters associated with CNN with Bi-LSTM.

A machine learning model's overall performance may be significantly enhanced using a loss function. During the training and validation phases, the loss is calculated, and its significance is determined by how well the model performs in each set. It is the sum of errors made in either the training or validation sets for each episode [50]. An accuracy measure is used to quantify the algorithm's performance in a useful way. The accuracy is usually estimated after the parameters that describe the model's correctness to the real true labels have been determined. Fig. 7 illustrates the hyperparameters, where the sub-figure (a) represents the model loss graphs and sub-figure (b) represents the accuracy graphs, and Table 2 provides an in-depth discussion of the environmental conditions used in this investigation. Both of these are provided in the context of the current research. The graphs show that the model has shown decent behavior without excessively fitting to the training data.

**Fig. 8:**
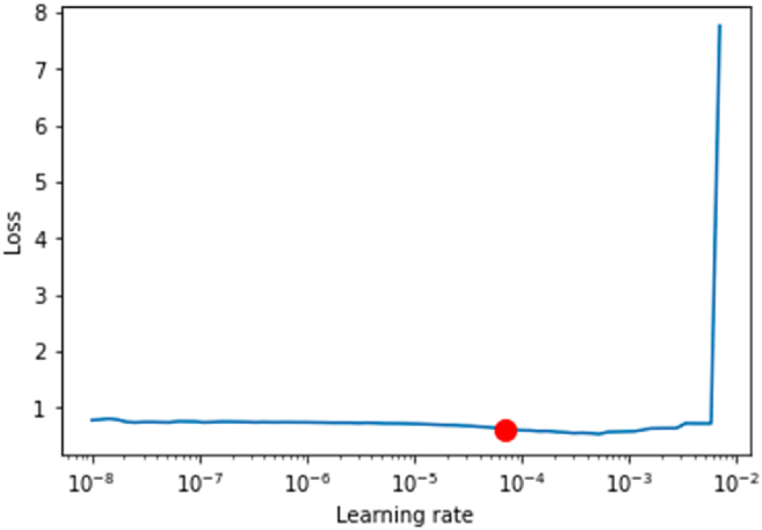
Learning rate graph of CNN with Bi-LSTM.

The learning rate of a deep learning model is extremely important in assessing the model's performance. In a very low learning rate, the model's training process is relatively slower in adjusting the associated weights in the network. Similarly, a higher learning rate might result in deviation from the desired outcome. The optimal learning rate limit is attained at the model's first point of divergence. When selecting the ideal learning rate, the loss should ideally continue to decrease at this point. The ideal learning rate of the Bi-LSTM with CNN model is highlighted with a red dot over the learning rate curve shown in Fig. 8.

### 5.5 Implementation environment

The proposed model is implemented on a stand-alone computer working in a Windows environment. The details of the implementation platform and the packages used in its evaluation process are presented in Table 3.

**Table 3:** Details of Experimental setup.

Environment Parameters	Specifications
Machine OS	Windows 11 64-Bit
Processor	Intel core ™i7-8550U CPU @ 1.99 GHz
Memory	8 GB DDR3 RAM
Implementation Language	Python
Implementation Platform	PyTorch, DL
Packages	Numpy, pandas, Scikit-learn, sklearn, Flask

### 5.6 Model explainability using SHAP

**Fig. 9:**
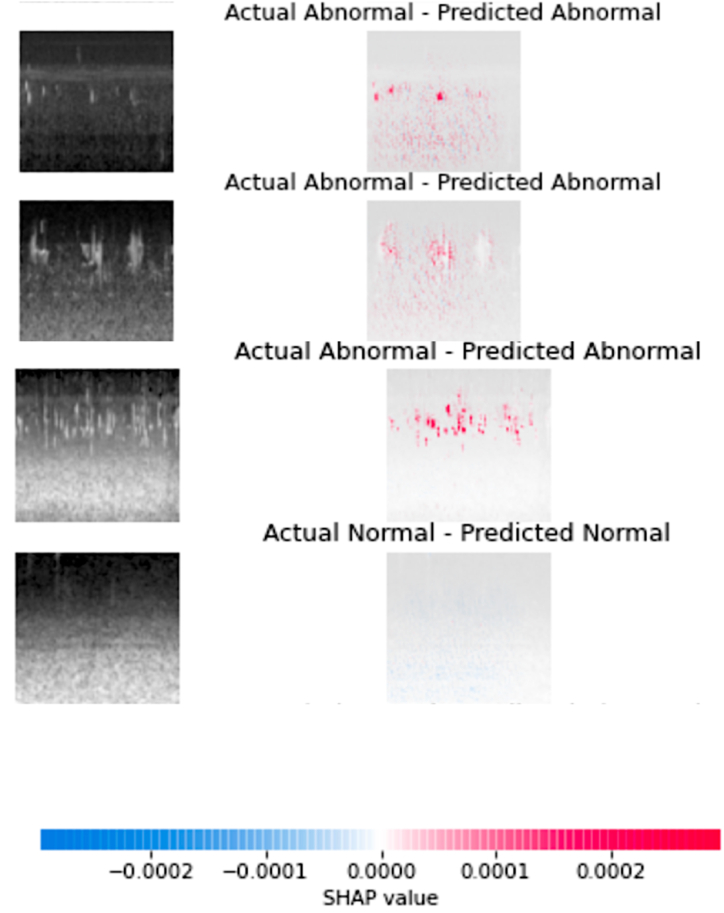
The SHAP values that are associated with the input data.

The model explainability is exceptionally significant in healthcare studies like tumor identification, healthcare informatics, and real-time monitoring to ensure the AI model's transparency and trustworthiness in the classification process. In the current study, Shapley values are used to explain the significance of the feature in making the prediction. Shapley values measure the feature's prediction contribution. They explain how input characteristics affect model prediction. The black-box models, like deep learning mechanisms, need to make the feature contribution explainable. The Shapley values associated with the spectrogram images are shown in Fig. 9.

Spectrogram images visually depict glucose dynamics across time, highlighting patterns that may not be apparent from raw data alone. SHAP values provide a way to recognize each input feature's contribution to the prediction model's output, allowing for a more granular understanding of model predictions. In the context of spectrogram analysis, SHAP values can help identify which features or frequency components in the images are most influential in predicting glucose levels. This increased interpretability may help discover biomarkers, comprehend physiological processes, and eventually improve diabetes treatment options. Furthermore, the openness afforded by XAI approaches, such as SHAP values, is critical in establishing confidence in AI-powered healthcare systems.

In dependency graphs of SHAP values, pink nodes represent features that positively affect predictions, while blue nodes represent features that have a negative impact. This color scheme helps visualize how different features contribute to model predictions. This gradual shift in colors visually illustrates the varying degrees of influence that features have on model predictions, allowing for a clearer understanding of the complex relationships within the data. The SHAP method effectively demonstrates its value in combining model-agnostic and model-specific justifications. By considering both the cumulative imputed values and the characteristics in the input vector. All possible feature combinations, including and excluding the feature set, must be checked for correctness before calculating the Shapley value. The number of potential combinations has also increased significantly.

As indicated by Equation (29), the explainable model's results are affected by the set of characteristics to which it is coupled. Independent variables m and n in the range up to x, are utilized to estimate the interdependence among the features. The Shapley value that is approximated is represented through the θ(m) notation.

(29) $$\theta \left(m\right)=\left\{\frac{\delta m}{\delta n_{1}}+\frac{\delta m}{\delta n_{2}}+\ldots +\frac{\delta m}{\delta n_{x}}\right\}$$

The notation θ stands for the change in weights associated with each feature vector. Equation (30) illustrates the connection between the feature vector and weight changes.

(30) $$\delta m=\left[\omega_{1}\times n_{1},\omega_{2}\times n_{2},\ldots ,\omega_{x}\times n_{x}\right]$$

The XAI models are considerably important in determining the feature significance in making the outcome. Shapley values provide insights into how individual characteristics affect the overall forecast by quantifying the marginal effect of each input variable, enabling transparency and interpretability.

## 6 Results and discussion

**Fig. 10:**
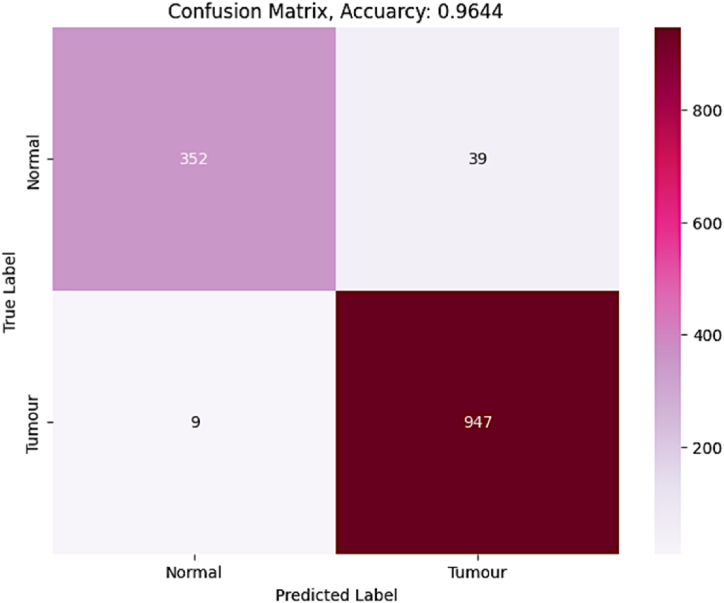
Confusion Matrix associated with CNN with Bi-LSTM.

The CNN with Bi-LSTM model performance is measured over the underlying facts linked with the spectrogram images. Other studies have used CNN with Bi-LSTM in the healthcare domain studies as EE-Based Emotion Recognition [51], schizophrenia detection using MSST-spectral images [52], epileptic seizure detection [53]. Similarly, there are various other domains in which the CNN with Bi-LSTM has outperformed in classification. The suggested CNN with the Bi-LSTM model is being assessed by measuring the model's true positive, true negative, false positive, and false-negative predictions. Based on the assessments, measures such as sensitivity, specificity, accuracy, and recall are calculated. Proper prediction of normal glucose level is termed true positive, while correct detection of aberrant glucose level is considered true negative. Similarly, misinterpreting excessive glucose levels as normal glucose levels results in a false positive, whereas misinterpreting normal glucose levels as abnormal glucose levels results in a false negative. Fig. 10 depicts the confusion matrix linked with the predictions.

The performance evaluation measures like sensitivity, specificity, accuracy, and F1-score are assessed from the generated confusion matrix [54,55]. The performances of various other state-of-the-art models concerning the above-discussed metrics are presented in Table 4. The comparison includes various approaches like Small-World Feed Forward Artificial Neural Network (SW-FFANN), ResNet18, ResNet50, SVM, Adaptive neuro-fuzzy inference system (ANFIS), Logistic Regression(LR), and Gaussian support vector machine (GSVM). The comparison is made over divergent datasets like PIMA, photoplethysmographic (PPG), Real-time sensor data, and electrocardiographic (ECG) signal data. The N/A in the table indicates the data is not available. The CNN with Bi-LSTM models has exhibited a reasonable accuracy compared to the other models used in the same field of study.

**Table 4:** Table representing the performances of various State-of-art models.

Approach	Dataset	Sensitivity	Specificity	Accuracy	F1-Score
SW-FFANN [56]	PIMA	0.85	0.96	0.91	N/A
ResNet18 [55]	PIMA	0.88	0.66	0.80	0.85
ResNet50 [55]	PIMA	0.94	0.57	0.80	0.85
SVM [55]	PIMA	0.95	0.83	0.90	0.93
SVM + RF + MLP + ANFIS [57]	Real-time sensor data	N/A	N/A	0.90	N/A
Deep transfer learning [58]	OhioT1DM dataset	0.59	0.98	0.95	0.61
LR [59]	PPG Signal data	0.73	0.64	0.69	N/A
GSVM [60]	PPG Signal data	0.79	0.83	0.81	N/A
Bayesian classifier [61]	PPG Signal data	1.00	0.87	0.93	N/A
SVM [62]	PPG Signal data	0.98	0.96	0.97	N/A
AdaBoost [63]	ECG Signal data	0.92	0.88	0.90	N/A
IGRNet [64]	ECG Signal data	0.80	0.77	0.77	N/A
GoogLeNet [64]	ECG Signal data	0.69	0.83	0.75	N/A
AlexNet [64]	ECG Signal data	0.77	0.82	0.74	N/A
HoG with K-NN [64]	ECG Images	0.70	0.79	0.76	N/A
AlexNet [19]	Spectrogram images	0.93	0.96	0.95	0.92
ResNet [19]	Spectrogram images	0.93	0.96	0.95	0.92
KNN [12]	Spectrogram images	0.89	0.89	0.86	0.86
SVM [12]	Spectrogram images	0.91	0.91	0.89	0.89
DenseNet-121 [65]	Spectrogram images	0.72	0.73	0.98	N/A
ResNet with SVM [66]	Spectrogram images	0.98	0.98	0.98	0.98
Proposed Approach	Spectrogram images	0.97	0.96	0.96	0.93

**Fig. 11:**
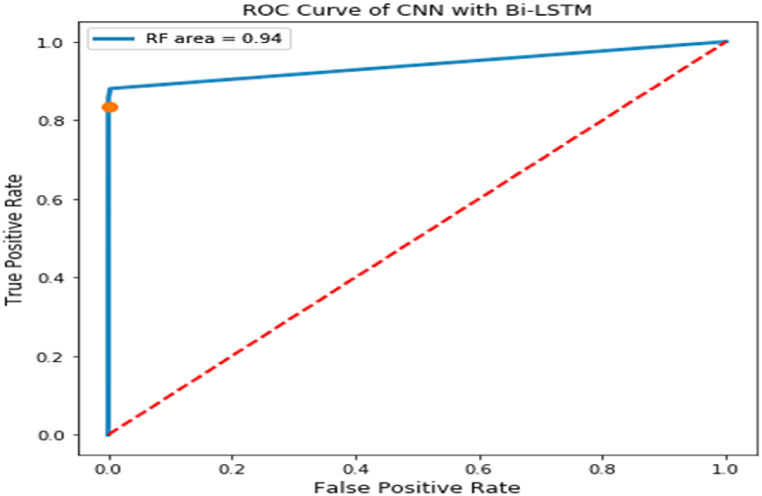
ROC curve associated with CNN with Bi-LSTM.

It can be depicted from the tabulated values shown in Table 4 that the efficiency of the CNN with Bi-LSTM performs well analyzed with the other robust models in the classification of diabetic individuals. Moreover, the Bi-LSTM with CNN model works with real-time spectrogram images rather than tabular datasets like PIMA. The ROC curve is a graph-based representation of the trade-offs between false and true positive rates. The X-axis usually presents the false-positive numbers, and the Y-axis presents the true-positive values. A true-positive rate closer to 1 is desired. The ROC curve of the CNN with the Bi-LSTM model is presented in Fig. 11.

It can be depicted from the ROC curves that the CNN with Bi-LSTM model has proven to have a fair and better performance in classifying the spectrogram images associated with abnormal glucose levels. Cross-validation (CV), is one of the many performance assessment measures that may be used to classify issues. CV operates by folding the data into numerous folds and guaranteeing that each fold is used as a test dataset. Its single value parameter specifies the number of folds, or groups that should be created from each test data sample before it can be validated. As a result, we call this procedure fold cross-validation. The results of applying the model's evaluation to several folds are shown in Table 5.

**Table 5:** Accuracy of the Bi-LSTM model concerning multi-fold cross-validation.

k value	Accuracy
2	0.964
3	0.951
4	0.956
5	0.959
6	0.969
7	0.972
8	0.979

Results show that the models' performance has continuously increased across the data samples in the assessment procedure. The diagnostic Odds Ratio (DOR) is a measure used to evaluate the effectiveness of a certain prediction associated with illness. DOR is calculated by dividing the likelihood that the test will return a positive result if the patient has an abnormal glucose level by the probability that the test would produce a positive result if the patient has a normal glucose level [67].

(31) $$DOR=\frac{True\;\left(positive\times negative\right)}{False\;\left(positive\times negative\right)}$$

In the current study, the DOR obtained is 949.6 on a standalone direct execution of the model. A test with high specificity and sensitivity and a low occurrence of false positives and false negatives has a higher DOR. DOR rises when test specificity improves, even while the test's sensitivity remains constant.

### 6.1 Future perspective model

**Fig. 12:**
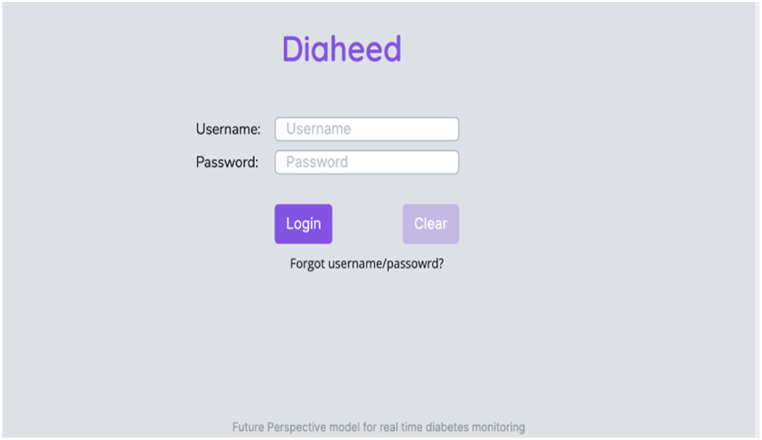
Agent authentication page of future perspective model.

**Fig. 13:**
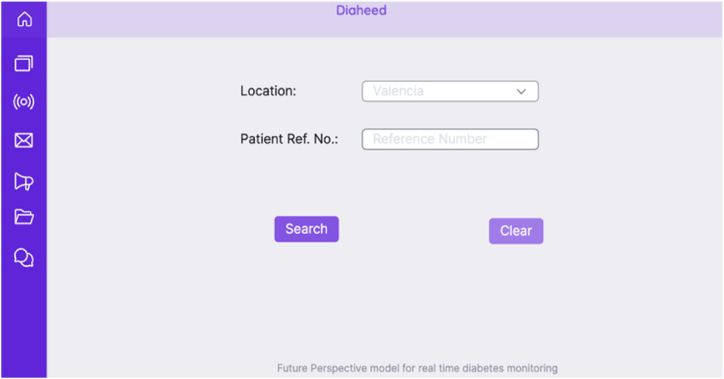
The patient search page of the future perspective model.

**Fig. 14:**
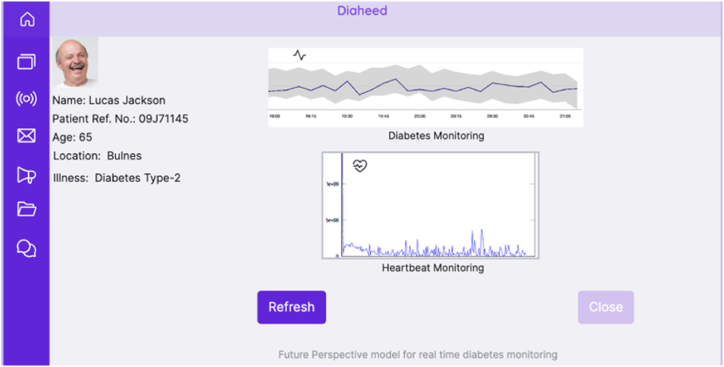
Patient dashboard of the future perspective model.

The future perspective model is a web-based application, where the caretakers are given a provision to access the real-time blood glucose levels and the heartbeat rate of the patients that are part of the ambient assisted community. The application would provide them the ability to access real-time healthcare data, update patient-related information, notify the patient individually or as a group, perform multi-patient monitoring simultaneously, and provide them with access to the messages that are sent by the patients. The chatbot facility would allow the future perspective model to have a friendly conversation with the patients for better convenience. The application is a Flask web framework that relies on the Jinja template engine to interface the business logic in the backend with the web interface for the user interface. NoSQL MongoDB [68] is used to save information on users and the data being tracked on them. The model is provided with user authentication to avoid unauthorized access to sensitive data. Fig. 12 shows the authentication interface to the application, and Fig. 13 shows the patient search page, where the user profile is searched based on the reference number of the patient. Fig. 14 shows the patient's dashboard, where the real-time monitoring details of glucose level and heartbeat rate are shown along with other basic information.

The future perspective model would rely on the spectrogram images generated from the sensor signal attached to the patient's body. The model's performance largely depends on the quality of the spectrogram image. The other potential challenge associated with the body sensor-based diabetes assessment from the spectrogram images includes the time frame between the spectrogram images and the length of the spectrogram images. The images are stripped to a confined length, impacting the model's performance. The current study uses spectrogram images of length 5sec to make predictions. However, more statistical analysis is needed on the length of the spectrogram images to ensure the accuracy of the prediction. The size of the spectrogram image is exceptionally significant in determining the prediction accuracy, as the larger spectrogram images can hold more signal data, which would result in better analysis of the underlying patterns in the data more precisely. The size of the spectrogram images is confined and fixed throughout the experimental analysis, which could be considered as one of the potential limitations of the current study. The hyperparameter tuning could yield a better accuracy [69], which was not done in the current study is also a potential limitation of the study.

## 7 Conclusion

The current study primarily categorizes the spectrogram images with abnormal glucose levels. By recognizing them, the model intends to notify the caretakers about the situation and provide timely medication and treatment. The CNN with Bi-LSTM model has performed well in precisely classifying the spectrogram images, making it unique. Considering the data samples as two classes, i.e., normal and abnormal glucose levels, the model has obtained a reasonable accuracy of 96.44 %. The findings demonstrate Bi-LSTM's ability to execute sequential data models and retrieve context data through forwarding and backward correlations over the dependencies from feature sequences. Compared to baseline approaches, the Bi-LSTM-driven model is more productive and accurate in classification quality. The XAI techniques would better assist in making the decision model more interpretable to the stakeholder. The model has outperformed the previous models in the current study concerning accuracy and F1-measure findings. The future perspective model would assist the caretakers in monitoring the patients remotely for a better monitoring experience.

The data in the current study is considered to be two classes, which could be considered as three classes, i.e., normal, low, and high blood glucose levels. So that the caretakers are given more appropriate information about the patient's condition, which would result in better medical outcomes. The acquisition of spectrogram images is a tedious task, and they are obtained at fixed time intervals, which could be considered one of the major technical limitations of the current study. The images of shorter time intervals could result in better outcomes. In the future, designing a model with minimal computational latency is necessary as the model is deployed over a real-time scenario to assist the patients in a much better way. Ablation studies are needed for problems such as diabetes prediction, which is considered to be the limitation of the results section of the current study. The ablation study could be considered in future research for better analysis of the performances of the model. Patient-specific sub-class training and classification would result in better results. The hyperparameters in the current study are decided based on the existing studies, and fine-tuning of the hyperparameters could result in better accurate results. The optimization of the hyperparameters is one of the future research directions of the current study. The multi-class problem with three classes, normal, low, and high blood glucose levels, is considered binary, i.e., normal and abnormal blood glucose levels. Multi-class analysis of the blood glucose levels could be fruitful and can be considered as a future research direction.

## Funding

This work was supported by the Deanship of Scientific Research, Vice Presidency for Graduate Studies and Scientific Research, King Faisal University, Saudi Arabia [Grant No. 5633].

## Ethical approval

The authors declare that they have no ethical approval required for this article.

## Data availability statement

The authors declare that the research data will be available for readers on request.

## CRediT authorship contribution statement

**Parvathaneni Naga Srinivasu:** Writing – original draft, Software, Methodology, Data curation, Conceptualization. **Shakeel Ahmed:** Supervision, Software, Investigation, Formal analysis, Data curation. **Mahmoud Hassaballah:** Writing – original draft, Validation, Resources, Project administration, Investigation, Conceptualization. **Naif Almusallam:** Validation, Supervision, Resources, Funding acquisition, Formal analysis, Data curation.

## Declaration of competing interest

The authors declare that they have no known competing financial interests or personal relationships that could have appeared to influence the work reported in this paper.
